# Impact of key parameters involved with plant-microbe interaction in context to global climate change

**DOI:** 10.3389/fmicb.2022.1008451

**Published:** 2022-09-30

**Authors:** Bharti Shree, Unnikrishnan Jayakrishnan, Shashi Bhushan

**Affiliations:** ^1^Department of Agricultural Biotechnology, College of Agriculture, Chaudhary Sarwan Kumar Himachal Pradesh Krishi Vishvavidyalaya, Palampur, India; ^2^Centre for the Environment, Indian Institute of Technology Guwahati, Guwahati, Assam, India; ^3^Department of Agriculture and Biosystem Engineering, North Dakota State University, Fargo, ND, United States

**Keywords:** climate change, environmental events, greenhouse gas emission, plant-microbe-environment interactions, salinity, temperature

## Abstract

Anthropogenic activities have a critical influence on climate change that directly or indirectly impacts plant and microbial diversity on our planet. Due to climate change, there is an increase in the intensity and frequency of extreme environmental events such as temperature rise, drought, and precipitation. The increase in greenhouse gas emissions such as CO_2_, CH_4_, NOx, water vapor, increase in global temperature, and change in rainfall patterns have impacted soil–plant-microbe interactions, which poses a serious threat to food security. Microbes in the soil play an essential role in plants’ resilience to abiotic and biotic stressors. The soil microbial communities are sensitive and responsive to these stressors. Therefore, a systemic approach to climate adaptation will be needed which acknowledges the multidimensional nature of plant-microbe-environment interactions. In the last two scores of years, there has been an enhancement in the understanding of plant’s response to microbes at physiological, biochemical, and molecular levels due to the availability of techniques and tools. This review highlights some of the critical factors influencing plant-microbe interactions under stress. The association and response of microbe and plants as a result of several stresses such as temperature, salinity, metal toxicity, and greenhouse gases are also depicted. New tools to study the molecular complexity of these interactions, such as genomic and sequencing approaches, which provide researchers greater accuracy, reproducibility, and flexibility for exploring plant-microbe–environment interactions under a changing climate, are also discussed in the review, which will be helpful in the development of resistant crops/plants in present and future.

## Introduction

Burning fossil fuels and widespread deforestation in the neoteric era have caused elevated atmospheric greenhouse gas (GHG) concentrations, and these changes in GHG have resulted in significant global climate shifts (Orlowsky and Seneviratne, 2012) also commonly known as global climate change. According to a recent IPCC report, the world has very little time before global climate change becomes troublesome (Gautam et al., 2021). Climate change has already warmed the planet: from the preindustrial period (1850–1900) to the present (1998–2018), the global average temperature over land has increased by 1.41 (Shukla et al., 2019). When GHGs are present in suitable concentrations in the earth’s environment, they trap radiation that the planet emits and prevent it from escaping back into space, keeping the planet warm enough to support life. Water vapor, carbon dioxide (CO_2_), methane (CH_4_), nitrogen oxides (NOx)_,_ and ozone (O_3_) are the primary greenhouse gases that trap energy and function as temperature regulators for the earth. Climate change over the years has threatened almost every individual on the planet, such as humans, plants, microbes, animals, and ultimately affecting their association, biogeographical cycles, food cycle, food security, etc., (Pecl et al., 2017; Rojas-Downing et al., 2017; Kumar and Verma, 2018; Caminade et al., 2019; Jansson and Hofmockel, 2020; Kasperson et al., 2022). The primary concern to food security is a decrease in crop productivity due to the rapid rise in global change in climate, as there is a downfall in crop production with every increase in degree Celsius (Rogelj et al., 2016). Given the lack of space to cultivate more land, it is prudent to monitor the remaining fertile land to regulate agricultural production closely, assure economic development, conserve biodiversity, and satisfy the ever-increasing food needs of the world’s population.

The significance of microorganisms in enhancing the nutritional bioavailability of plants is an essential climate-smart agriculture management technique and has been known for ages (Hamilton et al., 2016; Verma et al., 2017; Kumar and Verma, 2018; Jansson and Hofmockel, 2020). Several research studies have revealed advantageous interactions among plants, microbes, and the environment (Hamilton et al., 2016; Rosier et al., 2016; Liu et al., 2020; Romano et al., 2020). Root exudates are responsible for the abundance of microorganisms surrounding the root zone of crops and plants. They supply nutrients to the microorganisms promoting plant development through various growth-promoting characteristics. For example, plant growth-promoting rhizobacteria (PGPR) and mycorrhizal fungi are renowned for their capacity to promote plant development in stressful situations (Bach et al., 2016; Rêgo et al., 2018; Fattahi et al., 2021). Mycorrhizal fungi create symbiotic relationships with the majority of the crops/plants (Chun et al., 2018; Sangwan and Prasanna, 2022), which assist the agroecosystems in growing by improving nitrogen fixation (Hack et al., 2019; De Novais et al., 2020), synthesize bioactive compounds (Silva and Silva, 2020; Shah et al., 2022), boost photosynthesis (Jabborova et al., 2021; Bouskout et al., 2022), increase phosphatase activity (Metwally et al., 2021), and make osmotic adjustments under stress (Abd El-Samad and Abd El-Hakeem, 2019; Amjad et al., 2021), all of which help marginalized soils become more productive, detoxify metals, and increase resistance to both biological and abiotic stresses. By limiting plant pathogens in stressful environments, microbial interactions constitute close contact with the host plants and improve plant health (Lata et al., 2018; Trivedi et al., 2020; Sharma, 2021). Due to unpredictable climate change, the plant faces stress such as acidic soil, water deficit, salinity, osmotic stress, high temperature, low temperature, flooding, and an increase in biotic stress (Nazir et al., 2018) as a result of the change in soil condition (Mekala and Polepongu, 2019; Muluneh, 2020), that directly or indirectly affect its overall growth and development (Zhou et al., 2020; Malhi et al., 2021). Extreme habitats are one-of-a-kind ecosystems that support a diverse array of microbes, such as acidophilic, alkaliphilic, halophilic, psychrophilic, thermophilic, and xerophilic (Verma et al., 2017). However, interactions between plants and microbes need a suitable environment for their association and exchange of nutrients. This review will highlight the importance of microbial community for crops/plants and their interactions under stressful conditions arising due to global climate change, and how different abiotic stresses such as temperature, drought, salinity, elevated greenhouse gases, and heavy metal pollutants affect microbial association with plants. Understanding how traditional and emerging techniques can be used to understand the molecular complexity of plant-microbe interaction will enrich our knowledge in mitigating losses due to global climate change.

## Global climatic change and its implications on plant-microbe interaction

**Figure 1:**
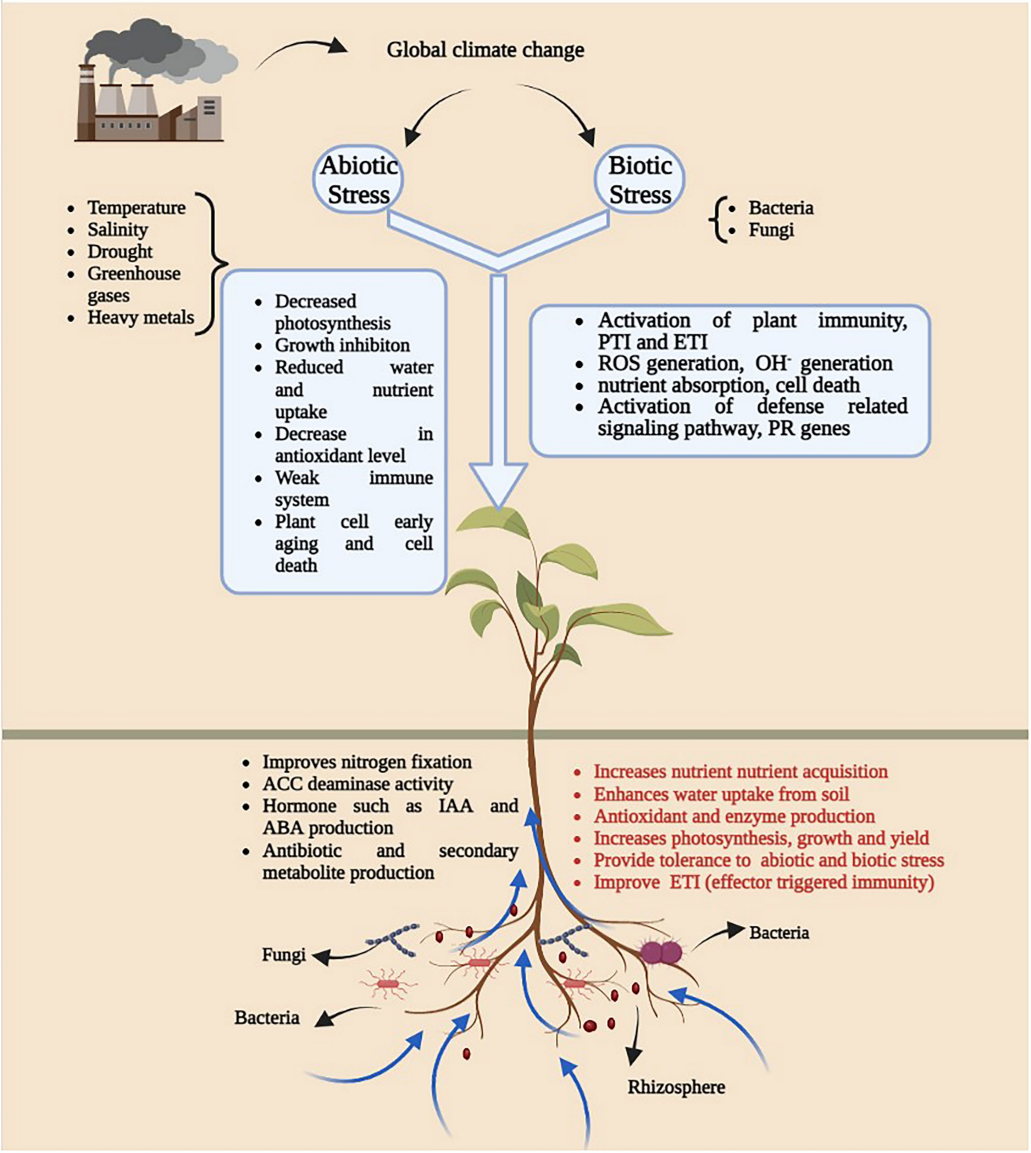
An overview of diagram illustrating the impact of global climatic change on plant-microbe interaction. Generation of abiotic and biotic stress as a result of climate change leads to several growth and development issues (White square box) in plants and microbes. Presence of plant growth promoting microbes in soil have positive impact on plant growth.

Climate change has imposed severe stress on plants accelerating microbial community disturbance and the spread of diseases, thereby increasing the management costs for techniques needed to mitigate and confront this global challenge. The association and interaction of microbe(s) and plants depend on the external environment, and any environmental perturbance will not be productive for either plants or microbes (Figure 1). In this review, we will focus on how environmental parameters are considered to influence molecular interactions between plants and microbes (Table 1).

**Table 1:** Impact of climate change induced stress on plant-microbe interaction.

Stress	Stress content^*^	Plant species	PGPM	Stress response in PGPM	Stress response in plant^#^	Plant growth parameter improved	References
Salinity+ mineral dust	NaCl (600 mM) + 1.5 g/ m^2^ month^1^	*Seidlitzia rosmarinus*	*Bacillus pumilus* HR *Zhihengliuella halotolerans*	Auxin, siderophore, ACC deaminase	Catalase activity, anthocyanin and decreased malondialdehyde, Na^+^ uptake	Chlorophyll a, protein, biomass, seed quality index, Fe, Mg, Mn content	Zilaie et al. (2022)
Salinity	NaCl (200 mM)	*Glycine max*	*Bipolaris* sp. CSL-1	Indole acetic acid, gibberellins, organic acids	Lower stress response gene expression, antioxidant Increased salicylic acid	Chlorophyll and shoot-root length, dry weight,	Lubna et al. (2022)
Heavy metal	Cr^6+^ (75 mg/l) + Cd^2+^ (200 mg/l)	*Sesbania sesban*	*Bacillus anthracis* PM21	ACC deaminase, IAA, EPS	Improved antioxidant enzymes activity, decreased proline content, electrolyte leakage, malondialdehyde content	Seed germination percentage, root length, shoot length, photosynthetic pigments	Ali et al. (2021)
Heavy metal	Cd (150 μg/ml)	*Oryza sativa*	*Colletotrichum* sp.	IAA, gibberlic acid, bioaccumulation, phosphate solubilization, siderophore	n.a	Root-shoot length, seedling biomass, chlorophyll, carotenoid content	Mukherjee et al. (2022)
Greenhouse gase + heavy metal	*e*CO_2_ (800 μl/l) Cd^2+^ (10 μM)	*Sedum alfredii*	*Bacillus megaterium*	n.a	Increased antioxidants, Cd^2+^ uptake, root to shoot transloaction	Shoot length, plant biomass, increased photosynthetic efficiency, altered rood exudate	
Drought	>10% PEG	Ryegrass	PGPR strains, *Bacillus* sp. WM13-24 and *Pseudomonas* sp. M30-35	More ACC deaminase activities and formation of mucoid colonies	Promoted growth and root development *via* regulating plant hormone and increased drought tolerance	Hormone distribution regulation, chlorophyll content, nitrogen and phosphorus contents	He et al. (2021)
Drought, temperature and heavy metal	1 mM of Ni, Cd, and Al each and 10% polyethylene glycol (PEG, 8000 MW) and 45 °C	*Glycine max* L.	Endophytic fungi LHL10 and LHL06	Accumulation of Ni, Cd, and Al, increased production of IAA	Mitigated metal accumulation and translocation, down-regulating heavy metal ATPase gene, drought-related and heat shock protein 90	Antioxidant activity, ABA and JA increased	Bilal et al. (2020)
Drought	water potential (0.0, −2.8, −4.8, and − 8.5 Ψ)	*Solanum lycopersicum*	*Trichoderma*	Nutrient availability	Higher shoot weight ratio	Increased tolerance to drought	Rawal et al. (2022)

IAA, Indole-3-acetic acid. EPS, Extracellular polymeric substance.

* Stress exposed to plants,

# Stress response post PGPM treatment.

### Effect of high temperature on plant-microbe interaction

The plant response is a complicated web of signalling that includes transcriptional networks and hormonal interactions that may be triggered by at least two different types of microbial signals; pathogen-associated molecular patterns (PAMPs) triggered immunity (PTI) and effector-triggered immunity (ETI). PAMPs include conserved patterns in bacteria and fungi, such as flagellin and chitin from bacteria and fungi, respectively (Kieser and Kagan, 2017; Taghavi et al., 2017; Zhai et al., 2018). PAMPs are recognized by the microbe leading to a basal level of defense in plants. However, this type of immunity of plants is usually suppressed by a virulent factor, also known as an effector (Dodds and Rathjen, 2010). Effectors recognized by plant signals are sent through the nucleus by nucleotide-binding leucine-rich repeat (NBS-LRR) receptors which ultimately activate effector-triggered immunity in plants (Saijo and Loo, 2020; Desaint et al., 2021). Global climate change and increase in temperature (any degree beyond optimal growth temperature) stamp out the ETI, leaving plants with weak immunity. This is a matter of great concern as most crops/plants rely on ETI for their growth, protection, and development (Cheng et al., 2013; Velásquez et al., 2018; Desaint et al., 2021). Even a short exposure to high-temperature changes the expression of genes of the effector-triggered immunity pathway. For example, the salicylic acid pathway was hindered when the temperature was relatively higher, leading to more susceptibility and infection by *Pseudomonas syringae* pv. *tomato* (Pst; Huot et al., 2017). The expression of genes and transcription factors of defense-related pathways are altered due to elevated temperature (Zhang et al., 2018; Wang et al., 2019). The decline in ETI, increased photorespiration, and disruption of photosynthesis gave rise to oxidative stress due to over production of reactive and non-reactive oxygen species. Thereby, plants upregulates various enzymatic and non-enzymatic antioxidant synthesis to counteract temperature induced stress (Awasthi et al., 2015).

Microbes also have a temperature range for optimal growth, reproduction, and infection; therefore, they are severely influenced by global climatic change. Beyond the optimal range, microbes become inactivated or inhibited, suppressing their plant growth promoting or infecting characteristics unless they quickly adapt to the temperature change (Velásquez et al., 2018). However, some microbes are specialized to survive under extreme environmental conditions and support other plant communities by faster carbon allocation (Heinemeyer et al., 2006). These specialized microbes are evolved to grow optimally under higher and lower temperatures or tolerate extreme temperature shifts without getting inhibited. Specialized enzymes and an elaborate network of secondary metabolites (extremolyte/compatible solutes) like carbohydrates, polyols, amino acids, etc., and their derivatives assist microbes in evading the negative influence of extreme temperature (Raddadi et al., 2015). A thermophilic *Klebsiella* sp. with the optimal temperature at 60°C produced siderophore, indole acetic acid, and ACC deaminase during normal growth (Mukherjee et al., 2020). *Thermomyces lanuginosus* is one of the most abundant fungi found in agricultural waste/compost, having an optimal temperature range of 45–50°C consisting of thermostable enzymes (Kumar et al., 2017).

It is anticipated that in a particular plant-microbe relationship, both plants and microbe will be subject to the effects of the extreme environmental conditions due to climate change. Plant disease occurs when both microbe and plant are oriented in a phenological manner and the risk of disease occurrence also shifts whenever a climate change is perceived by either plant or microbe (Figure 1). In this way, one can predict the possible outcomes of climate change on plant disease occurrence or whether the microbes will shift its niche to a broader environment due to resistance causing the disease to other plant groups (Ramsfield et al., 2016; Grace et al., 2019; Garrett et al., 2021). Some microbes, like arbuscular mycorrhizal fungi and endophytic bacteria, change their morphology to counteract the negative impact of elevated temperature in plants. A thermotolerant bacterial strain, *B. cereus SA1* produces components such as indole-3-acetic acid, salicylic acid, and gibberellin, leading to increased chlorophyll content and the biomass of soybean plants under high temperature stress (Khan et al., 2020b). Similarly, a facultative thermophile, *Klebsiella* sp., having a temperature range of 18–65°C, can also adjust to fluctuating temperatures with the simultaneous secretion of plant growth promoting factors. The inoculated *Oryza sativa* plants showed twice the length and 18 times more total grain mass yield than the control plants (Mukherjee et al., 2020). Similarly, the hyphal structure of mycorrhizal fungi changed with a rise in degrees celsius and transformed from having more vesicles at low temperatures to more hyphal networks at higher temperatures (Hawkes et al., 2008). A recent study showed that inoculating a mixture of *Paecilomyces formosus* LHL10 and *Penicillium funiculosum* LHL06 provided tolerance against stress induced by a combined high temperature (45°C)-salinity-drought condition. The inoculation promoted plant growth and photosynthetic activity, enhanced micronutrient uptake, reduced lipid peroxidation, and upregulated antioxidant activity (Bilal et al., 2020). These result in better carbon allocation to the rhizosphere and improved resistance (Heinemeyer et al., 2006; Compant et al., 2010).

The negative effects of temperature stress due to global climate change on plants may be reduced by the microbial community surrounding it, as it increases the range of temperatures at which the host plant grows. For example, when grown separately, tropical grass (*Dichanthelium lanuginosum*) and microbe *(Curvularia protuberate)* cannot grow at higher temperatures. However, under a close association, they show a symbiotic relationship and grow adequately, providing tolerance to heat (Márquez et al., 2007). A similar pattern of heat tolerance was observed in tomato plants when *C. protuberate* fungus was present in the soil (Rodriguez et al., 2008), suggesting the importance of microbe interaction with plants to enhance tolerance. Meanwhile, some microbes help cope with multiple stresses in plants, such as the bacteria *Burkholderia phytofirmans* PsJN strain that improve tolerance to heat (tomato), salinity and freezing (*Arabidopsis*), low temperature (grapevine), drought (wheat) stress (Miotto-Vilanova et al., 2016; Issa et al., 2018) and also possess antifungal characteristics (Miotto-Vilanova et al., 2016).

### Effect of salinity on plant-microbe interaction

Current estimates state that 7% of the total world land area (1.1 × 109 ha) is affected by salinization (Bayabil et al., 2021). Several environmental/anthropogenic factors cause an increase in soil salinity. Plants absorb the water-soluble salts formed by weathering of minerals. However, insufficient precipitation prevents the salts’ leaching, leading to the accumulation of soluble salt in the rhizosphere (Shrivastava and Kumar, 2015). Besides, saltwater intrusion through surface or groundwater connections can significantly influence soil salinity (Bayabil et al., 2021). Salinity stress in soil arises from the disruption of the soil’s ionic balance due to excess cations like Na^+^, Ca^2+^, K^+^, and anions like Cl^−^, NO_3_^−^. This ensues ion toxicity and osmotic imbalance irreversibility reducing plant growth and development. Moreover, high salt content disrupts water and nutrient uptake from surrounding soil due to osmotic stress. These effects induce oxidative stress due to the excessive generation of reactive oxygen species (ROS; Isayenkov and Maathuis, 2019). In addition, it affects the nodulation process, crop yield and reduces nitrogen fixation (reduction of nitrogenase; Kumar and Verma, 2018). Ion homeostasis, osmolyte accumulation, antioxidant regulation, polyamine mediated tolerance, nitric oxide assisted tolerance, hormonal regulation, etc., are some of the mechanisms adopted by plant or through plant-microbe interaction to impart resistance to adverse impacts of salinity stress (Gupta and Huang, 2014). Carrot plant varieties exposed to salinity stress by applying 100 mm NaCl for 22 days to maintain a topsoil salinity of 3.0 dS/m noticed significant biochemical changes like elevated ROS scavengers like glutathione, and ascorbic acid, the decline in reduced to oxidized glutathione ratio, and increased levels of osmoprotective proline (Kamińska et al., 2022). In another case, salt stress induction on a two-week-old *Arabidopsis thaliana* seedling by applying 150 mm NaCl for 5 days saw a 4 fold increase in alternative oxidase expression. The oxidase overexpression ensures better growth, reduced reactive nitric oxide, and ROS detoxification. Meanwhile, elevated superoxide, H_2_O_2_, lipid peroxidation, etc., imparted salinity tolerance for alternative oxidase silenced seedlings (Manbir et al., 2022). Meanwhile, plants innate defense mechanism in response to increased salinity initiates strong biochemical measures in halophytes than in others. Increasing the salinity of *Crithmum maritimum*, halophyte, from 200 to 500 mm NaCl spiked amino acids like glutamate, glycine, and tyrosine, while diminished serine, lysine, alanine, leucine, etc., content in foliar cells. A rise in the foliar concentration of phenolic compounds like 3-caffeoylquinic acid (64%) and saturated fatty acids like C16:0, C18:0, C20:0, and C22:0 resulted from increasing salinity. Besides, the interaction of salinity with nutrient limitation induces higher unsaturated fatty acids than saturated fatty acids. An increase in unsaturated to saturated fatty acid ratio is a crucial response against salinity stress as unsaturation increases plasma membrane permeability and fluidity (Castillo et al., 2022). Similarly, the proline, flavonoid, glycine betaine, anthocyanin, ascorbic acid content, and catalase-peroxidase activity increased at the expense of chlorophyll, biomass, and protein content up on exposure to *Seidlitzia rosmarinus* (desert halophyte) against salinity (0, 300, and 600 mm NaCl) and mineral dust (0 and 1.5 g/m^2^ month; Zilaie et al., 2022). On the contrary, all the markers for plant resistance to salinity stress showed a downward plunge in many other plants like *Pisum sativum,* indicating eventual necrosis (Gupta et al., 2021). Meanwhile, in some instances, exposure to salinity stress can be advantageous depending on the duration of exposure, severity, genotype, and plant development stages. Some studies have suggested that exposure to salinity has improved plant fertility by increasing clonal and sexual reproduction (Gupta et al., 2022).

Halophiles are groups of microbes that have optimum growth in wide range of NaCl concentration, like extremely halophilic (2.5–5.2 M), moderately halophilic (0.5–2.5 M), slightly halophiles (0.3–0.5 M) and halotolerant (<0.3 M) through several adaptive mechanisms (López-Ortega et al., 2021). Salt-in strategy excludes Na^+^ ions from cytoplasm through Na^+^/H^+^ antiporters and influx of K^+^ into the cytoplasm to balance the osmotic pressure. The salting-out strategy synthesizes and accumulates compatible solutes (trehalose, glutamate, ectoine, glycine, etc.) that act as stabilizers in the cell against stress (stabilize biological structures). Microbes also adopt antioxidant (enzymatic/non-enzymatic) responses against oxidative stress resulting from extreme salinity. These mechanisms are widespread in bacteria to survive other extreme conditions (Liu et al., 2019). Extracellular polymeric substance (EPS) production under salinity stress is a protective covering having mass transfer restrictions, retain water to avoid possible desiccation, and contains biological agents like nucleic acids, enzymes, exopolysaccharides, displays a protective role against salt incursion into a strong network of cells acting in unison against the stress (López-Ortega et al., 2021). These highly salinity resilient microbial communities spread across genera *Halomonas*, *Halothermothrix*, *Halobacillus Pseudoalteromonas*, *Arthrobacter*, *Vibrio*, *Salipiger*, *Chromohalobacter Streptomyces Bacillus*, *Viribacillus*, *Nesterenkonia*, and many more genera with fewer halophilic members (Liu et al., 2019; López-Ortega et al., 2021). A 6% NaCl content triggered EPS production in *Tetragenococcus halophilus* isolated from soya sauce moromi with 52.7% recovery (both fractions) consisting of glucose, galactose, mannose, and glucuronic acid (Zhang et al., 2022). Similarly, *Chromohalobacter japonicus* isolated from a rock salt waste accumulated ectoine, trehalose, N(4)-acetyl-l-2,4-diaminobutyrate, alanine, valine hydroxyectoine, and glutamate (compatible solutes) in the presence of 5% NaCl (Ananina et al., 2021). However, high salinity of 25% NaCl at pH 10.07 induced carotenoid pigmentation (0.98 g/l) in *Natrialba* sp. M6, found in salt lake water and sediments, possesses anticancer and antiviral activity (Hegazy et al., 2020). Similarly, fungi identified from hypersaline environments like water and sediments of salterns fall under genera *Hortaea*, *Phaeotheca*, *Aureobasidium*, *Trimmatostroma*, *Cladosporium*, *Aspergillus*, and *Penicillium*. The mechanism of halotolerant fungi aligns with that of halophilic bacteria/archaea, with the predominant tolerance route varying between species (Chung et al., 2019). A recent investigation on the salinity stress response of *Aspergillus sydowii* under hypoosmotic (0 mm NaCl) and hyperosmotic (2.0 mm NaCl) conditions revealed the accumulation of compatible solutes like glycerol, trehalose, arabitol, and mannitol (Rodríguez-Pupo et al., 2021).

The mostly investigated plant growth-promoting microbial (PGPM) species fall under genera like *Pseudomonas*, *Flavobacterium*, *Rhizobium*, *Acetobacter*, *Bacillus*, *Azospirillum*, *Aeromonas*, etc., (Etesami, 2020), arbuscular mycorrhizal fungi genera like *Funneliformis*, *Rhizophagus*, *Glomus*, *Claroideoglomus*, etc., (Diagne et al., 2020), and ectomycorrhizal fungi genera like *Amanita*, *Paxillus*, *Laccaria*, *Hebeloma*, *Pisoli* (Thiem et al., 2020). Salt tolerant PGPR confers the additional survival capacity to plants through nitrogen fixation, essential enzymes, phytohormones, solubilization of micro/macronutrients, plant pathogen inhibition, etc., (Etesami, 2020). Halophiles/halotolerant are an important class of PGPMs with immense plant growth promoting capacity. Such a group of strains composed of *Halomonas pacifica*, *Halomonas stenophila*, *Bacillus haynesii*, *Bacillus licheniformis*, *Oceanobacillus aidingensis*, etc., sequestered from coastal regions of Saurashtra, Gujarat, India exhibited nitrogen fixing, indole acetic acid production, phosphate-potash solubilization ability, and ACC deaminase activity (Reang et al., 2022). Similarly, PGPM communities colonizing the rhizosphere (endophytic) activate defensive mechanisms against increased salt concentration and stabilize their growth (López-Ortega et al., 2021). This allows PGPM to maintain the plant growth-promoting characteristics even under high salt content and stimulate the plant’s salinity resistance. This synergistic plant-microbe interaction under the influence of salinity results in a cumulative stress response enabling better or normal growth during plant salinity stress. Meanwhile, PGPMs without salt tolerance severs mutualistic association with plants due to cell death/senescence or preference for their survival like spore or cyst formation (Etesami, 2020). The synergistic effect improved the stress response in *Seidlitzia rosmarinus* inoculated with *Zhihengliuella halotolerans* indicated by increased levels of Mg^2+^ (63%), Fe^2+^ (45%), Mn^2+^ (21%), Na^+^ (53%), chlorophyll (40%), biomass (35%), seedling quality index (104%), and protein (48%). Interestingly, the presence of PGPM decreased the plant’s secondary metabolite content in response to stress by promoting growth. PGPM prevents cellular damage by terminating ROS production, reducing allied scavenging metabolite production in plants, and promoting growth-related factors (Zilaie et al., 2022). Meanwhile, PGPM’s interaction with non-halophytes confers stress defense and promotes plant growth. ACC deaminase positive *Bacillus marisflavi* and *Bacillus cereus*, inoculated to *Pisum sativum* seedlings exposed to salinity (1% NaCl), showed alleviated levels of crucial parameters of plant like reducing sugars, biomass, phenols, flavonoid, chlorophyll content, and antioxidant enzyme levels (Gupta et al., 2021).

A PGPM consortium of *Bacillus* sp., *Delftia* sp., *Enterobacter* sp., *Achromobacter* sp. showing phosphate solubilization and siderophore production, indole acetic acid, and ammonia inoculated to *Solanum lycopersicum* showed enhancing effect on plant growth and stress response against salinity (Kapadia et al., 2021). Plant growth promoting endophytic bacterias like *Curtobacterium oceanosedimentum SAK1, Curtobacterium luteum SAK2, Enterobacter ludwigii SAK5, Bacillus cereus SA1, Micrococcus yunnanensis SA2, Enterobacter tabaci SA3* has shown to increase biomass growth of *Waito*-C rice under slat stress (150 mm NaCl) due to increased glutathione, and sugar content. The salt tolerance was augmented by improved expression of flavin monooxygenase and auxin efflux carrier (Khan et al., 2020a). The fungi-plant interaction also exhibited increased growth, antioxidant activity, and compatible solute accumulation under salinity stress (Diagne et al., 2020). *Euonymus maackii* Rupr exposed to salinity stress (50-200 mM NaCl) reduced nutrient uptake, photosynthetic capacity, and morphology but stimulated the antioxidant system and salt ion accumulation. Meanwhile, treatment with *Rhizophagus intraradices* showed improved superoxide dismutase, peroxidase, and catalase activity, plant growth, chlorophyll content, nutrient uptake, and reduced ion accumulation (Li Z. et al., 2020). Similarly, *Alnus glutinosa* Gaertn inoculated with ectomycorrhizal fungus *Paxillus involutus* OW-5 showed improved growth and proline content under a moderate salinity of 50 mM NaCl (Thiem et al., 2020). In many cases, fungal-plant interaction also decreases the plant’s defensive mechanism by stimulating plant growth and reducing salt stress. *Glycine max* seedling primed with endophytic-growth promoting fungus, *Bipolaris* sp. CSL-1 decreased plant’s stress-related gene expression and improved plant growth by reducing salt stress through ion homeostasis (Na+/K+ exchange), bioaccumulation, ion translocation, etc., and fungal phytohormone secretion upon exposure to 200 mM NaCl stress (Lubna et al., 2022). Similar results were returned by applying endophytic fungus *Porostereum spadiceum*-AGH786 to *Triticum aestivum* at 140 mM NaCl stress (Gul et al., 2022).

### Effect of heavy metal pollutants on structure and function of the microbe and its interaction with plants

Heavy metal pollution is a serious environmental issue which spread across the globe. Metals and metalloids with a density ≥5 g/cm^2^ causes heavy metal pollution. These fall under two groups: toxic metals – arsenic, cadmium, copper, nickel, chromium, cobalt, lead, mercury, tin, and zinc, precious metals – gold, platinum, ruthenium palladium, and silver, and radionuclides – americium, radium, thorium, and uranium. The leakage of excess metals into soil and water transpires due to biotic factors like weathering of rocks, leaching from metal ores in soil, atmospheric deposition, or anthropogenic sources like mining, electroplating, dye and pigment manufacturing, battering production, tannery, and other sources generating or employing metals for various processes (Devi et al., 2022). In soil, metal ions are an important abiotic factor for the proper growth of plants by supplying essential micronutrients. Heavy metal ions under minimum inhibitory level fail to elicit any significant negative implications on plant metabolism. However, bioaccumulation of these heavy metals to toxic levels leads to interference in metabolic pathways, distortion of biomolecules (DNA, RNA, protein), cell wall destabilization, etc., that triggers cellular defence mechanisms like ROS (Nazli et al., 2020). Leaf chlorosis, protein degradation, lipid peroxidation, etc., ensues from the plant’s oxidative response. Plants raise similar morphological and biochemical mechanisms to defend against heavy metal toxicity as microbes. Morphological modifications act as the first line of defence like cell wall alteration, thick cuticles, and trichomes formation. The biochemical mechanism overtakes toxicity resistance by increasing metal toxicity and paves the way for intracellular metal accumulation (Nazli et al., 2020).

The biochemical mechanism attempts to detoxify, reduce, immobilize, efflux, etc., heavy metals with the assistance of various biomolecules. Metal chelators like nicotianamine, citrate, proline, glutathione, metallothioneins, phytochelatins, flavonoids, caffeic acid, and quercetin enable extracellular and intracellular sequestration promoting metal speciation for biochemical processing. Various transporter systems like cation diffusion facilitator, ATP-binding cassette, cation antiporters, natural resistance-associated macrophage, heavy metal ATPases, etc., are involved in ion homeostasis of essential metal ions and efflux of a toxic level of heavy metal (Viehweger, 2014). This transporter system assists in heavy metal accumulation, compartmentalization in inert form, and translocation into roots and shoots, negating the toxicity effect (Hassan et al., 2022). Metallochaperones play a pivotal role in the spatial relocation of metal ions in plant cells for further process according to relevance. ROS generation by redox imbalance in plants or directly by heavy metals can cause irreversible damage to the cellular components. Enzymatic and non-enzymatic antioxidants act as scavengers to quench the effects of oxidative stress. Phytohormone production (jasmonic acid, ethylene, abscisic acid, etc.) is another strategy adopted by plants to circumvent heavy metal toxicity by promoting cellular growth or as signal transduction molecules to trigger a specific defence mechanism (Viehweger, 2014). Cd^2+^ stress on *Spinacia oleracea* negatively impacted biomass growth, chlorophyll content, and gas exchange attributes. However, plant stress response elevated hydrogen peroxide, proline accumulation, ascorbic acid content, malondialdehyde, and enzymatic antioxidant activity. Meanwhile, foliar application of peptone enhanced plant growth and photosynthesis due to lower Cd^2+^ uptake, reduced oxidative stress response, and increased antioxidant activity (Emanuil et al., 2020). Similarly, *Capsicum annuum* supplemented with 40 mg/l Cr^4+^ generated a moderate level of stress response like growth inhibition, rise in malondialdehyde content, etc. The addition of 24-epibrassinolide observed a significant reaction against heavy metal toxicity and Cr^4+^ accumulation. Many genes related to auxin signaling, glutathione mechanism, MAPK pathway, ABC transporters, and other stress-related genes were upregulated, leading to better stress response, leaf architecture, root growth, and chlorophyll content. Moreover, the root accumulated more Cr^4+^ than leaves due to the regulation of metal transport gene expression (Mumtaz et al., 2022).

Many investigations have pointed out that heavy metal toxicity provokes DNA damage, protein denature, inhibits bacterial cell division and DNA transcription culminating in the loss of cell viability. Hence, microorganisms have evolved and adapted to survive and grow under heavy metal toxicity by adopting different morphological and biochemical upgradation. Heavy metal tolerance indicates the increasing limit till microbes cell retains its viability, where the tolerance mechanism can prevent metal ions from indulging detrimental damage to cell machinery (Syed et al., 2021). The morphological adaptation deters metal ions from entering the cytoplasm and induces a series of cellular defence mechanisms that could lead to apoptosis. For example, microbes modify cell wall composition to restrict the permeability of metal, like down-regulation of porin production to exclude Cu^2+^ from membrane ion channel. In addition, the functional groups available on the cell wall direct the adsorption and accumulation of metal ions, especially the carboxyl groups presented by proteoglycan. Due to this, gram +ve bacteria possess better heavy metal adsorption than gram −ve bacteria. Besides, EPS production in response to physiological stress has also been reported to accumulate heavy metals depending on the amount of anionic carbohydrate content (Nanda et al., 2019). Autoaggregation (same species) or coaggregation (different species) strategy also provides a cumulative response against metal toxicity. The aggregation followed by biofilm formation is observed in some microbial communities (Pal et al., 2022). However, cells are armed with biochemical adaptations when the metal ions traverse the cell wall. Extracellular efflux by ionic pumps encoded by bacterial plasmid confers resistance against toxic heavy metals like Sb^3+^ and Zn^2+^. Microbes have also exhibited heavy metal detoxification by complex formation using thiol-containing groups like metallothioneins, redox conversion as an electron acceptor or enzymatic transformation into less toxic oxidation forms (cytochrome C oxidase, mercuric reductase), and intra-extra cellular sequestration inside vacuoles (Nanda et al., 2019). Microbes secrets certain secondary metabolites like siderophore, oxalate, phosphate, sulfate, etc., as chelating agents for extracellular sequestration in a steady state heavy metal concentration. Enterobactin, yersiniabactin, pyoverdine, pyochelin, aerobactin, etc., are some of the microbial siderophores contributing to heavy metal resistance (Pal et al., 2022). Different genes encode a combination of these mechanisms to alleviate the toxic effects of heavy metals. The arsRDABC operon, CadA system, merRDTPA operon, cnrCBA efflux system, copABCD, czrCBA operon encode resistance for As^5+^/Sb^3+^, Cd^2+^, Hg^2+^, Ni^2+^/Co^2+^, Cu^2+^, Zn^2+^, respectively *via* reduction-efflux mechanism (Nanda et al., 2019). Biomethylation of metalloids, like dimethylselenium, during volatile compound formation followed by permeation across cell membranes also assists in metal resistance. Also, some investigations have found microbes to down-regulate chemotaxis proteins and cellular motility proteins for heavy metal resistance (Pal et al., 2022).

Rhizobacteria are primarily involved in the heavy metal resistance in soil. *Enterobacter cloacae* MC9 cultured from rhizosphere soil around *Capsicum annum* showed maximum resistance towards Cd^2+^, Cr^6+^, Pb^2+^, and Ni^2+^. *E.colacae* MC9 production of siderophores-salicylic acid, 2,3-dihydroxybenzoic acid, ACC deaminase, and EPS conferred heavy metal tolerance. However, as heavy metal concentration increases, the defensive mechanism weakens to the maximum tolerance limit, beyond which cell viability is lost due to respiratory inhibition. Increasing heavy metal content reduced the indole acetic acid production capacity of the rhizobacteria (Syed et al., 2021). Similarly, *Bacillus* sp. S3 cultured from soil collected from the antimony mining area showed potential multi-metal resistance capability against Cd^2+^, Cr^4+^, Cu^2+^, and Sb^3+^. The increasing metal ion concentration (toxicity) induced EPS production from *Bacillus* sp. S3 with increased protein content. This aromatic-like protein precipitates metal ions other than antimony accompanied by a certain degree of intracellular immobilization. Meanwhile, intracellular bioaccumulation and detoxification depicted Sb^3+^ resistance rather than EPS adsorption (Zeng et al., 2020). Fungi also have adapted resistance against metal toxicity, assisting in a strong mutualistic survival with plants in the heavy metal polluted rhizosphere (Văcar et al., 2021). *Trichoderma aureoviride* TaN16 found in the rhizosphere soil of rice plants was found to possess multi-heavy metal resistance against Cd^2+^, Ni^2+^, and Co^2+^ (Sarkar et al., 2022). Genetically modified *Pichia pastoris* with overexpressed cytochrome b5 reductase showed high resistance to Au^3+^ and Pd^2+^ through bioaccumulation and reduction into nanoparticles (Elahian et al., 2020). *Cladosporium* sp., *Didymella glomerata*, *Fusarium oxysporum*, *Phoma costaricensis*, and *Sarocladium kiliense* (Ascomycota) fungal species showed very high minimal inhibitory concentration for Hg^2+^ (140–200 mg/l) attributed to adsorption of metal ions to the cell surface and possible intracellular ingestion leading to a biosorption capacity of 33.8 to 54.9 mg/g dry weight. *Lecanicillium* sp., *Fusarium solani*, *Fusarium equiseti*, *Penicillium crustosum*, *Penicillium brevicompactum*, *Cadophora malorum*, *Stagonosporopsis* sp., and *Mortierella alpina* also indicated Hg^2+^ tolerance capacity (Văcar et al., 2021). *Komagataella phaffi* isolated from soil around a mine showed high resistance against Cr, Pb, Cd, and Cu (Liaquat et al., 2020). These metal removal characteristics in the form of bioaccumulation and adsorption make fungi ideal candidates for heavy metal bioremediation and alleviating the metal toxicity of plants.

In the event of heavy metal toxicity or gradual rise in toxic levels in soil, microbes with ability to avoid heavy metal stress initiate their resistance mechanism to normalize their growth compared to the surrounding environment. Similarly, heavy metal resistance protocols are activated in plants to facilitate growth and allied process. Microbes negative for heavy metal resistance can evolve the defence mechanism to an extent by adaptation or orthogonal gene transfer, while plants without stress tolerance could wilt away. Symbiotic association of resistant PGPMs with plants in the rhizosphere supply necessary plant hormones, improve nutrient availability, promote plant’s antioxidant response, and precipitate heavy metal away from plant tissues (siderophore) to sustain plant growth and mutualistic benefits for microbes (Kumar and Verma, 2018). Hence, PGPMs need to survive under metal toxicity to augment or impart stress tolerance to plants. *Leclercia adecarboxylata* MO1’s secretion of siderophores assisted the tolerance against Zn^2+^and allowed indole acid production that augmented the antioxidant enzyme activity, lipid peroxidation, glutathione content to minimize zinc toxicity in cucumber seeds (Kang et al., 2021). Meanwhile, some studies reported plant-microbe interaction to reduce the plant’s stress-related genotypic and phenotypic response by effectively quenching heavy metal toxicity and promoting growth (Jan et al., 2019). The type of heavy metal toxicity can modulate the PGPM’s mechanism for ensuring plant cell viability. Pb^2+^ and Cu^2+^ toxicity initiated considerable indole acetic acid production in *Bradyrhizobium japonicum* (nitrogen fixing) than a decline in Ni^2+^. Meanwhile, an increase in Cu^2+^ and Ni^2+^ showed a reduction in biomass growth. Inoculation of PGPM to lettuce plant showed a combined growth promotion and reduction in heavy metal bioavailability due to sorption on the cell membrane by amine and nitro group to impact the plant’s survivability under heavy metal toxicity (Seneviratne et al., 2016). *Bacillus cereus* inoculated to soil sowed with *Brassica nigra* seedling conferred the plant resistance to increasing Cr^3+^ with multiple defence mechanisms. The surge in osmotic adjustment (proline and sugar), antioxidant enzyme activities, bioaccumulation, and translocation led to increased plant growth (shoot, root length), biomass content, photosynthetic pigments, water status, etc., (Akhtar et al., 2021). The stronger heavy metal resistance of PGPM imparts stable interaction leading to higher survivability and proper plant growth. The potential multi-metal impedance of *Bacillus anthracis* PM21conferred proper growth and photosynthesis under Cd^2+^ and Cr^4+^ toxicity (Ali et al., 2021). Moreover, PGPM-plant interaction also improves the plant’s resistance against heavy metal toxicity by increasing bioaccumulation (inside cellular compartments) and translocation (root to stem), as observed for *Bacillus paranthracis* NT1 increasing Cd^2+^ uptake by 41.8% in *Solanum nigrum* (Chi et al., 2022). Similarly, the symbiotic relationship of fungus, *Rhizophagus irregularis,* and grass species *Brachiaria mutica* led to improved chlorophyll and protein content due to antioxidant-mediated resistance and bioaccumulation of Cr^6+^ toxicity (Kullu et al., 2020). Moreover, co-inoculation of arbuscular mycorrhizal fungus-*Glomus mosseae* and the rhizobacteria *Sinorhizobium meliloti* to alfa alfa showed a significant improvement in Cd^2+^ resistance than the influence of individual microbes. Co-inoculation increased antioxidant enzymes that mitigated the effect of lipid peroxidation and ROS (Wang et al., 2021).

Similar to rhizobacteria, endophytic PGPM plays a pivotal role in a plant’s survival against heavy metal toxicity. Endophytic bacteria, *Enterobacter ludwigii* SAK5, *and Exiguobacterium indicum* SA22 isolated from various plants growing near sea beach showed high Cd^2+^ and Ni^2+^ accumulation and elevated glutathione content for stress mitigation. Rice seed cultivars inoculated with the strains in hydroponic medium showed heavy metal accumulation in root than shoot. The endophytes prevented the toxic effects of heavy metals by reducing oxidative stress, stress response gene expression, and abscisic acid production. Meanwhile, biomass growth and chlorophyll content improved (Jan et al., 2019). Similarly, endophytic- photostimulation-root colonizing fungi, *Colletotrichum* sp. isolated from the leaves of *Eupatorium triplinerve* effectively reduced the bioavailability of Cd^2+^ during rice seedling germination through bioaccumulation and biosorption. This augmented plant growth, biomass, and pigment production under metal toxicity (Mukherjee et al., 2022). Inoculation of a consortium of such endophytic fungi to *Alocasia calidora* imparted resistance and growth against landfill soil contaminated with a mixture of heavy metals through the production of compatible solute, antioxidant, and heavy metal bioaccumulation (root and shoot; Hassan et al., 2022). Recent works have demonstrated biochar’s beneficial traits like high surface area for PGPM to attach, high carbon to increase soil organic matter, nutrient availability for the microbes, improved cation exchange efficiency, high water retention, etc., to improve plant’s toxicity resistance and heavy metal removal through plant-microbe interaction (Harindintwali et al., 2020).

### Influence of drought as a result of global climate on plant-microbe interaction

One of the identifiable impacts of climate change is high fluctuation in precipitation patterns influencing the moisture content in air and soil, leading to inundation or drought. Insufficient or lack of moisture, drought stress poses a greater threat to food security as varying exposure affects crop yield in different amplitude (Gupta et al., 2022). Water deficient conditions trigger a series of morphological and physiological changes in plants as an after-effect or to stabilize the growth (Kumar and Verma, 2018). In the early phase, drought stress reduces shoot growth while maintaining root growth resulting in an enhanced root/shoot ratio. Besides, severe drought wrinkles the plant cell wall, leading to the development of fewer leaves and reducing the plant’s fresh weight due to a fall in turgor pressure. The root morphology also changes (shrinking of roots) to alter water and nutrient allocation to various plant parts under exposure to drought stress to prevent dehydration that could force leaves to lose the ability to use photosystem II. Lack of water also affects the availability and absorption of nutrients through roots and relocation to other parts (Gupta et al., 2022). In addition, drought stress affects cell wall integrity, produces ROS, promotes early leaf senescence, increases ethylene production, decreases chlorophyll content, and reduces photosynthetic activity (Kumar and Verma, 2018). Moreover, salts released from soil accumulate in the rhizosphere without water to dissolve and translocate, giving rise to salinity stress (Gupta et al., 2022).

Certain plant varieties exposed to water scare situations for generations would have adapted genetical traits to survive and grow normally. The drought-stress tolerance evolved by these plants also use phytohormones to improve growth under limited water content, activate cellular mechanisms to efflux excess salt from plant cells and translocate it to other parts, produce compatible solutes to mitigate the effect of drought stress and maintain water balance, increase antioxidant enzymes/chemical to scavenge excess ROS generated, etc., (Gupta et al., 2022). Plants also take up physiological measures like altering stomatal conductance, root length increment, leaf rolling, shortened life cycle, hairy leaves, etc., to escape water scarcity or maintain water potential (Seleiman et al., 2021). The metabolomic approach revealed the enhanced production of glycine betaine, proline, sorbitol, mannitol, unsaturated fatty acids, tocopherol, ascorbate, and jasmonate conferred drought stress tolerance in *Thymus serpyllum* (Moradi et al., 2017). Moreover, modulating the abscisic acid (ABA) concentration is a primary plant response to drought (osmotic stress or water deficit). Any change in ABA initiates a signaling cascade that leads to extensive transcriptional modification, phosphorylation events, and physiological modifications in stomata, such as the closing of stomata to mitigate transpiration rate (Li S. et al., 2020; Soma et al., 2021). Some studies found that exogenous addition of ABA, glycine betaine, and glutathione imparted drought tolerance to plants by lowering oxidative stress, declining lipid peroxidation, stimulating compatible osmolyte, antioxidant enzymes, etc., (Nawaz and Wang, 2020; Sohag et al., 2020). The expression of transcription factors, like MYB, MAT, ERF, and CCR, has been found to effect stress-responsive genes in plants encoding a large spectrum of secondary metabolites (Yadav et al., 2021). Overexpression of gene encoding transcription factor, TaWRKY2 significantly enhanced the expression of stress responsive genes like *DREB1*, *DREB3*, *GST6*, *ERF5a*, *TaWRKY19*, and *TIP2* (Gao et al., 2018). Aquaporins are primary water channel proteins that transport water and neutral solutes across the membrane. Overexpression of tonoplast aquaporin intrinsic protein (*TaTIP4;1*) in *Arabidopsis* and rice enabled seedling germination and growth under drought and salt stress (Wang et al., 2022).

The scarcity or absence of water also impacts microbial diversity, cellular functions, and soil characteristics (Manzanera, 2021). Xerophilic microbes have been found to survive under low water activity (a_w_ < 0.7), the mole fraction of water, arising due to lack of moisture, desiccation from radiation, high salt concentration, etc. The a_w_ for pure water is considered to be 1 and it decreases with the increment of salts or decline in water content. Meanwhile, theoretical a_w_ minima for halophiles range between 0.611–0.632. These xerophiles become the initial colonizers on soil surfaces with low a_w_ that pave the way for extended harboring of life similar to planetary body surfaces that are under the influence of radiation (Merino et al., 2019). The primary defense mechanism of these microbes is xeroprotectants which are osmolyte/compatible solutes protecting the cell from an increase in salt and desiccation. These include sugars (trehalose, fucose, fructose, etc.), polyols and their derivatives (mannitol, glycerol, inositol, etc.), amino acid derivatives (ectoine, glycine, proline, etc.,), methylamine (glycine betaine), and certain DNA molecules. Ion homeostasis (to avert osmotic imbalance), heat shock response, antioxidant molecules, specific protein synthesis (dehydrin), water storing or attracting molecules like proteins and exopolysaccharides, indole acetic acid, cytokinins, ACC deaminase, volatile organic compounds, etc., are also involved in managing water deficient condition by microbes (Manzanera, 2021). A sugar-tolerant fungus, *Xeromyces bisporus* was reported to have the lowest limit of a_w_, ~0.605, exhibiting proper cell division. *Pseudomonas syringae*, widely transported within bioaerosol, possess a protein coating that freezes water at a warmer temperature that provides water as an internal thin film under exposure to short-wave radiation. *Pseudomonads* also secrets hygroscopic biosurfactants and alginate, increasing the bioavailability of water in the vicinity of the microbe (Stevenson et al., 2015). Microbial community analysis of a deep-sea hypersaline anoxic basin, the Kryos basin, at the seawater (MgCl_2_)-brine interface having a_w_ of ~0.4 found sulfate-oxidizing bacteria (likely *Desulfovermiculus* and *Desulfobacula*) and aerobic methanotrophs (Steinle et al., 2018). A recent investigation isolated *Bacillus* sp. and *Paenibacillus* sp. from rhizosphere soil of the Caatinga biome, Brazil grew under a_w_ of 0.919 by synthesizing exopolymeric substance, indole acetic acid, and ACC deaminase (Braga et al., 2022). Similarly, species belonging to taxa *Antarctomyces, Cladosporium, Mortierella, Leptosphaeria, Penicillium, Pseudogymnoascus, and Thelebolus* isolated from rhizosphere, roots, and leaves of Antarctic angiosperms were capable of normal growth at a_w_ of 0.81 and 0.66 (Coelho et al., 2021). Another study identified xerophilic *Penicillium michoacanense, Penicillium melanosporum,* and *Penicillium siccitolerans* in the soil samples from Spain and Mexico also grew normally at a_w_ of 0.76 (Rodríguez-Andrade et al., 2021).

Therefore, microbes associated with plants initiate their defense mechanism and stabilize their growth after exposure to drought stress. These microbes releases metabolites that build up the stress response and normal growth of associated plants by increasing the root/shoot ratio, developing more biomass, improving the water uptake capacity, improving the bioavailability of nutrients, increasing productivity, and resisting drought (Al-Karaki et al., 2004; Egerton-Warburton et al., 2008; He et al., 2021; Rawal et al., 2022). In return, the root exudates supply additional factors that influence the stress response of these microbes (Gupta et al., 2022). Bacteria such as *P.syringae* (having *flg*22 as PAMPs) can be sensed by receptor FLS2 resulting in the closure of stomata to prevent microbe entry in Arabidopsis as a result of ABA induction (Melotto et al., 2006). Moreover, elevated ABA leads to repression of the salicylic acid pathway in plants post-infection, and therefore plants have reduced ETI and tolerance (Jiang et al., 2010). Xu et al. (2018) examined the impact of soil moisture content on sorghum and concluded that there is a reduction in drought stress implication in the rhizosphere. Microbes like *Ralstonia solanacearum* are important in sensing the soil moisture content as the expression of two cell wall-related kinase genes (*WAK16* AND *WAK3*-*2*) was reduced and reflected a weaker immune in ginger plants (Jiang et al., 2018). Biofilm formation on roots and secondary metabolites secretion from xerotolerant rhizobacteria diluted serious implications of drought stress in *Glycine max* L, increasing root and shoot fresh weight (Braga et al., 2022). Indole acetic acid drained into the rhizosphere by *Rhodobacter sphaeroides* KE149 effected notable changes in drought stress tolerance in adzuki bean plants. The inoculation decreased endogenous ABA and jasmonic acid, increasing salicylic acid and proline content. A significant increase in Ca^2+^, Mg^2+^, K^+^ accumulation by lowering Na^+^ was also observed in the shoot region of the plant. These biochemical changes improved root length, shoot length, biomass weight, and chlorophyll content of adzuki bean plants (Kang et al., 2020). Another study conducted by Cheng et al. (2022) suggested that *Funneliformis mosseae* (Arbuscular mycorrhizal fungus) and trifoliate orange interaction too had a positive impact on plant growth and development as this interaction increased phenolics, terpenoid content and reduced alkanes, esters, and amides from the root exudates thus mitigating the effect of drought. A consortium of arbuscular mycorrhizal fungi belonging to genera *Glomus, Gigaspora, Acaulospora, and Entrophospora* inoculated into two carob ecotypes increased plant growth, stomatal conductance, photosystem II efficiency, water content, and mineral uptake along with a reduction in lipid peroxidation and oxidative stress after 4 days of recovery from drought stress (Boutasknit et al., 2020). The application of dual fungal-rhizobacterial application has also affected the physiological and morphological improvement in plants during drought stress exposure by abating oxidate damages and augmenting water/nutrient supply (Azizi et al., 2021). These findings must also be aligned with varying climate patterns to improve or sustain the current agroeconomic scenario.

### Effect of elevated greenhouse gases on plant-microbe interaction

Greenhouse gases spread layers of protective covering over the earth’s atmosphere, maintaining a surface temperature of 14°C. Carbon dioxide, methane, nitrogen oxides, and ozone contribute to the GHG that absorbs the infrared radiation reflected from the earth’s surface. Besides, the gaseous layer absorbs UV radiations and other potentially harmful radiation from reaching the biosphere (Cassia et al., 2018). At balanced GHG levels, the gases enter the plant *via* stomata and get integrated into the plant’s biochemical cycle, thereby fixing atmospheric GHGs into biomass and biomolecule. This leads to root exudates and plant carbohydrates benefiting rhizobial and endophytic microbes and strengthening plant-microbe interaction (Weyens et al., 2015). However, many natural and (mostly) anthropogenic events have destabilized GHG composition translating into a rise in atmospheric temperature at an alarming rate. The elevated GHG levels provoke climate changes like floods, drought, heat waves, etc., that exerts extreme stress and toxicity on plants and microbe, affecting the agroeconomy worldwide. Oxidative stress and ROS generation resulting from a rise in GHG levels and climate changes have severe implications for plants and associated microbes (Cassia et al., 2018).

Carbon dioxide occupies 76% of GHG emissions worldwide. Hence, increasing the level of CO_2_ poses grave implications compared to other greenhouse gases (USEPA, 2022a). The elevated CO_2_ levels (*e*CO_2_) can influence the plant-microbe interaction and their stress tolerance capacity. Many investigations have found *e*CO_2_ to directly translate into higher biomass production, nutrient utilization, photosynthetic rate, water consumption, and carbohydrate content. The improved photosynthetic conversion increases product allocation to roots, leading to branched roots and modifying root exudate composition. Besides, improved biomass growth enables higher heavy metal accumulation, extracellular sequestration by siderophore production, and other compatible solute production to mitigate rhizosphere stress factors (Rajkumar et al., 2013). Arsenic accumulation and severe oxidative stress ensued from exposure of barley toward. As toxicity indicated by a higher level of H_2_O_2_ and lipid oxidation under ambient CO_2_. However, *e*CO_2_ (620 ppm) improved the plant’s root/shoot dry weight and antioxidant system, alleviating As stress (AbdElgawad et al., 2021). These changes alter the diversity and activity of the rhizosphere microbial community. The influence on plant growth promoting microbial growth in the rhizosphere further promotes plant growth and stress tolerance. The endophytic microbes also benefit from the improved biomolecule contents of plants, enabling their growth and plant-promoting factor production (Rajkumar et al., 2013). Biofortification of *Sedum alfredii* Hance (hyper and non-hyper accumulator) with a combined *e*CO_2_ (800 μl/l) and endophyte inoculation of *Bacillus megaterium* sp. M002 under Cd^2+^ stress resulted in greater plant biomass, increased photosynthetic efficiency, decreased pH, organic carbon, nitrogen, and sugar content of root exudate, higher Cd^2+^ uptake, root to shoot translocation, and increased antioxidant enzyme content (Tang et al., 2019). Moreover, many studies have reported increased biomass content of soil microbes under high CO_2_ partial pressure (Singh et al., 2019). The atmospheric CO_2_ can dissolve in the moisture trapped within the soil particles followed by microbial uptake, thereby influencing microbial stress response. The *e*CO_2_ alleviated nCeO_2_ and nCr_2_O_3_ nanoparticle toxicity by enhanced microbial utilization of carbon, reduced nanoparticle availability (uptake), and metal resistance microbial selection. These conditions improved the soil’s microbial diversity by selecting metal-resistant microbes belonging to classes *Alphaproteobacteria*, *Gammaproteobacteria*, and *Bacteroidia* (Luo et al., 2020). Also, *e*CO_2_ could affect the biodegradation ability of soil microbes due to a decrease in nitrogen as a consequence of plant biomass growth and reduced carbon to nitrogen ratio (Singh et al., 2019).

Nitric oxide (NO), nitrous oxide (N_2_O), and nitrogen dioxide (NO_2_) make up the oxides of nitrogen involved in GHG emissions. The NOx in the atmosphere routes towards plants through foliar or rhizobial adsorption and stomatal translocation into the apoplast. These oxides act as one of the precursors of biochemical compounds (amines, amino acids) during photochemical reaction through the nitrate assimilation pathway, functioning as nutrition to plants (Weyens et al., 2015). Climate change induced plant stress, and increased NOx in the atmosphere could induce excess reactive nitrogen species (RNS) generation leading to oxidative stress detrimental to the plant’s photosynthetic activity and growth (Cassia et al., 2018). However, RNS also function as signaling molecules promoting legume-bacteria interaction for root nodulation, improving nitrogen-fixing capability, and regulating the transcription of genes encoding various nitrogenase activities (Signorelli et al., 2020). Similar to CO_2_, NOx can also be taken up by plant-associated microbes in the form of nitrite or nitrate and assimilated as a nitrogen source microbial cellular growth and maintenance (Singh et al., 2019). In microbes, flavoprotein and single-domain hemoglobin have been shown to detoxify RNS generated through nitrite assimilation. Some microbes resort to EPS production as a barrier against plant-generated ROS/RNS, as demonstrated by *Rhizobium leguminosarum* bv. *trifolii* against reactive species produced by clover plants (Signorelli et al., 2020). Hence, a stable plant-microbe interaction imparts additional tolerance against GHG and other climate change linked stress factors, delivering microbial growth and nutritional requirement in the form of exudate strengthening microbial survivability in the stressed ambiance.

Methane accounts for 20% of the world’s GHG emissions after CO_2_ and is 25 times more potent in trapping heat than CO_2_ (USEPA, 2022b). CH_4_ is emitted from many natural-anthropogenic sources, and wetlands account for 24% of this evolution. Plants-microbe interaction is a centerpiece in the plant’s role as a source or sink for CH_4_. Methane evolution from plants is related to symbiotic methanogenic microbes or vascular conduits for funneling the gas produced by soil bacteria (Stępniewska et al., 2018). Aerobic- nonmicrobial methane production from plants involves ROS generation during stress followed by converting precursor molecules like methyl groups, methionine, etc., into CH_4_ (Putkinen et al., 2021). Meanwhile, many plant-associated methanotrophic microbes oxidize atmospheric CH_4_ to CO_2,_ followed by integration into the photosynthetic channel. Such conversion has been found in methanotrophs like *Methylocella palustris* and *Methylocapsa acidiphila* colonizing *Sphagnum* sp. (Stępniewska et al., 2018). In addition, several endophytic and rhizobial methanotrophs belonging to genera *Methylocystis*, *Melthylomicrobium*, *Methylococcus*, *Methylomonas*, etc., can convert CH_4_ into molecules like ectoine, hydroxyectoine, glutamate, sucrose, lipids, EPS, 5-oxoproline, methanol. These bioactive molecules can assist plants and associated microbes in stress tolerance induced as a result of climate change (Sahoo et al., 2021). Hence, stronger stress tolerance of microbes renders improved tolerance of plants towards atmospheric methane and other impacts of climate change.

## Assessment of techniques involved in the investigation of molecular complexity of plant-microbe interaction

Most of our knowledge and understanding is limited to the influence of microbes on plant growth based on isolated bacteria or microbes examined under controlled conditions without considering the impact of soil condition, weather influence, abiotic stresses, and the response of these on microbes as well as soil. Plant growth promoting bacteria (PGPB) usually functions when present in close association with other consortia, microbial communities, plants, and soil (Glick, 2015). Microbiomes play a short-term role in determining plant adaptation to climate change, whereas microbiomes and their hosts form a long-term relationship in determining adaptation to climate change (Trivedi et al., 2022). Therefore, it is an essential of the hour to develop techniques, methods, and approaches to understand plant-microbe interaction under changing environmental conditions.

The OMICS-based approach primarily includes various techniques associated with genomics, transcriptomics, proteomics, metabolomics, and metagenomics (Hugenholtz and Tyson, 2008; Fadiji and Babalola, 2020). It expands the horizon of knowledge regarding functional, structural, ecological interactions, and the evolutionary history of individuals. Metagenomics is the recent method to determine the microbiota of soil where both plant and microbe interacts by directly taking the sample from soil and analyzing it through the nucleotide sequencing/DNA sequencing method (Hugenholtz and Tyson, 2008; Castro-Moretti et al., 2020; Fadiji and Babalola, 2020). Several studies such as soil nematode (cyst nematode) and its association with plant root in soyabean, rice root nematode association, bacterial interaction with plant root biome such as (*Ralstonia solanacearum*) and solanaceous crops was was highlighted using metagenomics approach to study microbe and plant interactions (Hu et al., 2017; Kang et al., 2018; Zeiss et al., 2019). The next generation sequencing method can be used to analyze a plethora of samples for the presence of different kinds of microbes in the soil and its association with the plant can be further studied using the transcriptomics-metabolomics approach (Sogin et al., 2006; Hajibabaei et al., 2011; Caporaso et al., 2012). The whole genome shotgun sequencing of *Brevibacterium frigoritolerans* near maize crops facing salt stress and drought stress revealed to possess proteins essential for coping with drought and salinity and improving tolerance and crop yield (Zhang et al., 2019). The importance of “pan-genome” study in understanding the role of all genes present in the microbial strain is also blooming and it has helped researchers in creating an artificial environment supporting plant life under stress conditions (Brockhurst et al., 2019; Costa et al., 2020; Golicz et al., 2020). As suggested by Segata et al. (2013), it is possible to study a whole complex ecosystem using transcriptomics, proteomics, and metagenomics, that includes not only the host plant but also consists of the surrounding environment (soil, temperature, pathogens). The transcriptomics-based approach includes RNA sequencing and gene expression analysis that emphasizes the importance of a particular gene under specific environmental conditions (Lowe et al., 2017). At present, to understand plant-microbe relations under specific conditions, RNA sequence based analysis is used predominantly on cultured microbes separately from plants revealing the significance of individual genes in relation to plant adaptability (Mirzaee et al., 2015; Yi et al., 2017). One example where a transcriptomics-based approach was used to study the impact of bacterial strain (*Bacillus subtilis*) on cucumber roots revealed differential expression of genes controlling signaling pathway (LRR, PR-4, ARG7, auxin response gene) as a result of biotic stress induction (Samaras et al., 2022). Proteomics and metabolomics are the studies of proteins and metabolites using techniques such as liquid chromatography, spectrophotometry, high performance liquid chromatography, and nuclear magnetic resonance spectroscopy (^1^H NMR).

GC/MS and LC/MS (gas chromatography, liquid chromatography mass spectroscopy) is used to estimate, identify, characterize the chemical components such as flavonoids, polyamines, spermidine, etc. that are released during plant microbe interactions (Rauha et al., 2000; Macoy et al., 2015; Zeiss et al., 2019). The basic steps include extraction of proteins from the sample, followed by isolation, characterization, analysis using spectroscopy, and comparing-generating a protein/metabolite database (Cheng et al., 2009; Levy et al., 2018). A most recent approach, also known as metaproteome, was used to analyze the bacterial community surrounding the vineyard. This can provide precise information regarding proteins involved in the stress response of plants (Novello et al., 2017; Bona et al., 2019).

Apart from the application of OMICS-based approaches, which is expensive and time consuming, imaging-based techniques like fluorescence microscopy, X-ray crystallography, microscopic techniques, and nanoscale secondary ion mass spectroscopy (NanoSIMS) can also provide a significant amount of information regarding plant-microbe interaction facing global climatic changes (Young and Crawford, 2004; Vos et al., 2013; Steffens et al., 2017). The prime requisite in this technique is to identify the individual strain with a marker and monitor the structure, function, and behavior of the microbe present in a complex environment (Steffens et al., 2017; Wilpiszeski et al., 2019). A combination of techniques like FISH (fluorescence *in situ* hybridization), mass spectrometry, and Raman microspectroscopy (Kumar et al., 2015) have been used to analyze the environmental samples under complex microbial communities (Musat et al., 2012; Kumar and Ghosh, 2019).

## Conclusion and future perspective

At present, identifying novel ways to increase global crop production for the growing human population is one of the most challenging tasks where plant stress and diseases pose a major threat to global food security. Research on plant diseases and plant immunity has made remarkable advances in the past couple of decades. Molecular and mechanistic insight into what drives plant-microbe interactions is still at a very primitive stage. This is because most of the experiments considering plant-microbe interactions are carried out in laboratory conditions or growth chambers that do not replicate the actual field conditions (static) faced by both plant and microbe as actual field condition keeps on changing due to global climate change. We have seen how plant and microbe interaction changes due to abiotic stresses caused by global climate change. The impact of high and low-temperature stress on changes in the structure of microbe, modification of gene expression, activity, and its relationship with plant roots was analyzed. Further, the impact of salinity, heavy metal, and drought on plant-microbe interactions as a result of changing environment revealed that microbes could have both positive and negative results on plant growth and development. We need to consider a multidimensional plant-microbe interaction that includes techniques such as metagenomics, NGS, and imaging techniques altogether to get more detailed information on the implications of changing environmental conditions. Abiotic and biotic stresses are equally problematic for crop plants, but research focused on plant-microbiome interactions promise for increasing their resilience and producing resistant crops.

## Author contributions

BS: conceptualization, methodology, visualization, investigation, resources, data curation, and writing-original draft. UJ: conceptualization, methodology, visualization, investigation, resources, data curation, and writing-original draft. SB: conceptualization, methodology, visualization, writing-review and editing.

## Conflict of interest

The authors declare that the research was conducted in the absence of any commercial or financial relationships that could be construed as a potential conflict of interest.

## Publisher’s note

All claims expressed in this article are solely those of the authors and do not necessarily represent those of their affiliated organizations, or those of the publisher, the editors and the reviewers. Any product that may be evaluated in this article, or claim that may be made by its manufacturer, is not guaranteed or endorsed by the publisher.
